# Data-Efficient and Fast Machine Learning Molecular Dynamics through Integrated Active Learning and Knowledge Distillation

**DOI:** 10.1021/acs.jctc.6c00917

**Published:** 2026-08-21

**Authors:** Xiliang Lian, Alfredo Pasquarello

**Affiliations:** Chaire de Simulation à l’Echelle Atomique (CSEA), 27218 Ecole Polytechnique Fédérale de Lausanne (EPFL), Lausanne CH−1015, Switzerland

## Abstract

We develop data-efficient machine learning interatomic potentials (MLIPs) for fast molecular dynamics simulations by combining DeePMD and MACE models within an active learning and knowledge distillation framework. Using liquid water as a case study, we first independently train DeePMD and MACE models from scratch through active learning. We find that MACE requires around 3 times less training data than DeePMD, but its inference speed is 10 times lower. We also show that starting from a pretrained foundation model based on the MACE architecture further reduces the training data by a factor of 7, resulting in a fine-tuned foundation model with a 25 times data reduction compared to DeePMD. To overcome the limitation associated with the lower inference speed of MACE potentials, we next develop a knowledge distillation scheme to train a DeePMD potential from the fine-tuned foundation model through an inexpensive active learning workflow. The distilled model is generated with ∼10 times less computer time than the DeePMD model trained from scratch, while showing the same fast inference speed. Comparison with *ab initio* calculations shows that all the models reach the same level of accuracy in reproducing structural, vibrational, and diffusive properties of liquid water. Our approach enables practical, data-efficient training of customized MLIPs with high speed and accuracy.

## Graphical abstract

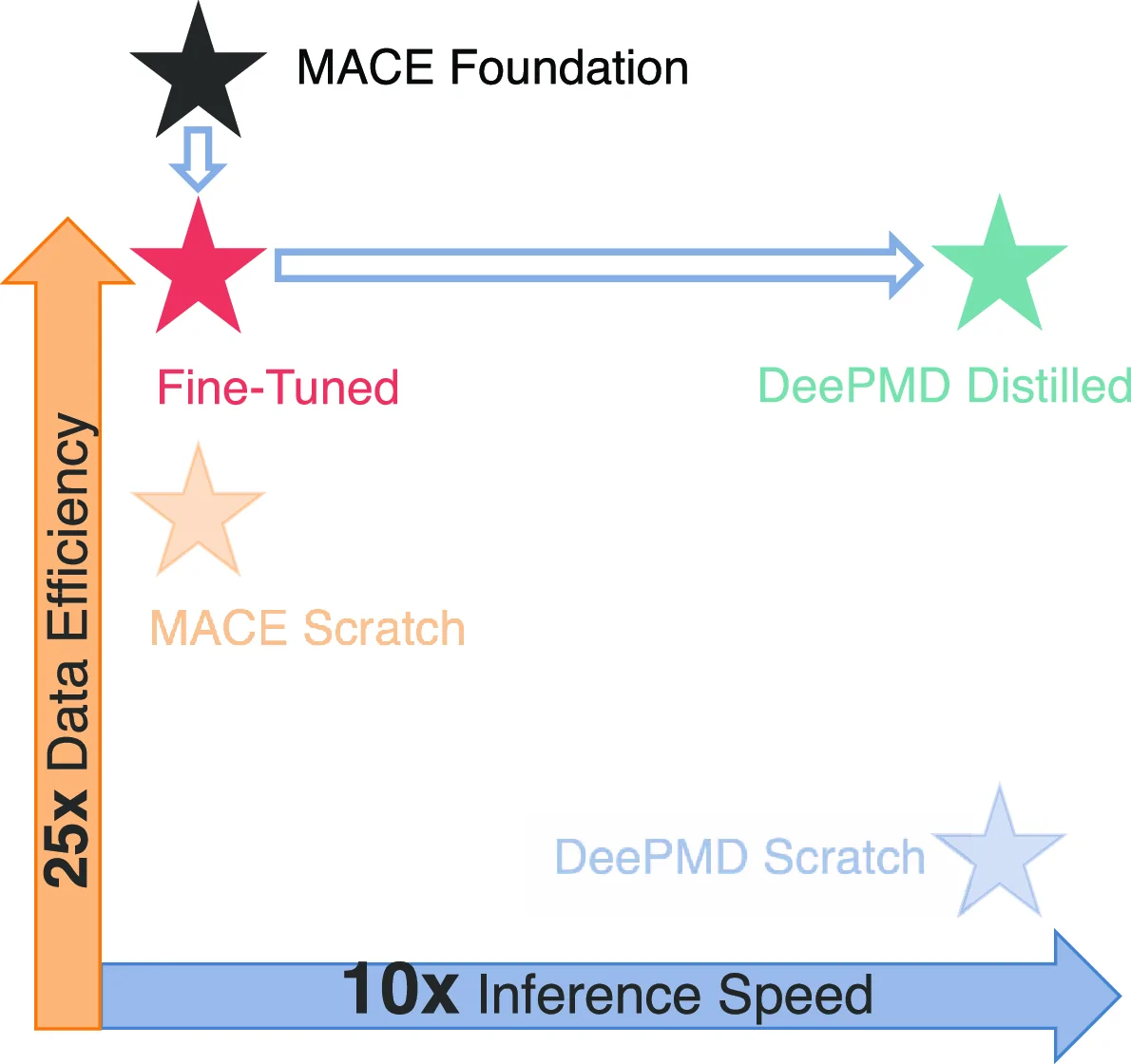

## Introduction

Machine learning interatomic potentials (MLIPs) have revolutionized molecular dynamics (MD) simulations by providing *ab initio* accuracy with efficiency comparable to that of classical force fields.^1–6^ Over the past few years, the motivation to develop more accurate and data-efficient models has driven the architectural evolution from local atomic descriptor-based methods, such as deep molecular dynamics (DeePMD), to graph-based MLIPs, such as MACE.^3,7,8^ This advancement has shifted the field from specialized models trained from scratch to ready-to-use foundation models or universal force fields, such as MACE-MP-0, DPA-2, and Orb-v3.^9–11^ These foundation models rely on the graph neural network architecture and are pretrained on databases covering a large set of chemical environments. They have showcased excellent extrapolability when applied to various systems, although discrepancies can occur.^9,10^ While this field has seen significant progress, improving the data efficiency and computational speed still underlies the application of MLIPs.

For a fixed model architecture and hyperparameters, the quality of the MLIP is fully determined by the training data. This data set consists of labeled structures computed using high-level *ab initio* calculations and should represent the desired region of the potential energy surface.^12,13^ Therefore, data acquisition critically determines the training efficiency. Two strategies are primarily used to acquire the training data.^14^ The most straightforward method is to directly extract information from *ab initio* molecular dynamics (AIMD) trajectories, along which configurations with energies and forces are collected. For liquid water, one of the most important and challenging systems, MLIPs based on a variety of architectures have been shown to accurately reproduce the structural, transport, and thermodynamic properties of liquid water via direct sampling.^3,12,15–17^ However, this strategy can be inefficient because AIMD simulations are expensive and neighboring configurations in the AIMD trajectory can be similar. Therefore, active learning based on kernel error estimation and on query-by-committee (QbC) has been proposed to prepare the data set more efficiently and in a more structured manner.^17–100^ MLIP-based MD simulations are performed, and errors are predicted to evaluate the model confidence, through which new data are collected or abandoned. The model gets iteratively updated with growing training data until it converges. Active learning using the QbC method has also been applied to train MLIPs for liquid water and has demonstrated excellent agreement with AIMD simulations.^19^ Active learning has been applied to a variety of systems, thereby demonstrating robust performance and generalizability.^20,21^

The development of foundation models represents a milestone in the MLIP field and could drastically reduce the efforts required for data acquisition. Ideally, with foundation models, no new training data is needed since these models have been pretrained with a vast amount of accurate data. In practice, refining them into specialized models with domain-specific knowledge or corresponding to specific energy functionals is often necessary.^22^ Such a specialization procedure is referred to as fine-tuning. In this case, the foundation models serve as stepping stones toward accurate and specialized models. With a small data set, the foundation model can then be fine-tuned to deliver results in excellent agreement with models trained from scratch.^22–24^ However, foundation models are generally based on complex graph neural networks. This results in low computational efficiency and hinders the practical application of fine-tuned foundation models (FT-FMs) to large-scale MD simulations.

Force fields that enable fast evaluation are central to MD simulations. For MLIPs, the performance is closely related to the underlying regression model, although other factors such as GPU acceleration, model compression, and parallelization also influence the inference speed.^4,25^ The computational speed can vary by an order of magnitude or more when going from descriptor-based to graph-based MLIPs.^17,26,27^ When considering accuracy, graph-based models combining equivariant message passing with efficient many-body messages usually demonstrate superior accuracy. However, the sophisticated architecture degrades computational speed. Descriptor-based architectures are typically less expressive but are more efficient than the heavily parametrized graph-based models, a category that includes the foundation models. Therefore, leveraging the advantages of the two approaches, namely, the inference speed of descriptor-based MLIPs and the expressivity of graph-based MLIPs, remains a significant challenge.

Knowledge distillation from computer science can be borrowed to accomplish this task.^28,29^ This methodology allows a simpler student model to be taught by a more complex teacher model. The student model then mimics the behavior of the teacher model without carrying its complexity. There are examples where these concepts have been used to generate MLIPs,^30,31^ but a full integration within active learning workflows has yet to be realized.

In this work, we develop accurate and efficient MLIPs for molecular dynamics by introducing a distillation scheme in which a descriptor-based model is derived from a graph-based foundation model. Using liquid water at the hybrid functional level as a target system, we first train DeePMD and MACE models from scratch. For these, we determine the required number of *ab initio* calculations as well as the inference speed. Next, we consider two sequential steps for further improvement. In the first step, we show how the data efficiency can be boosted by adopting a foundation model and fine-tuning its parameters through active learning. In the second step, DeePMD-based MLIPs with a high inference speed are achieved through a distillation process in which the fine-tuned foundation model is used as a surrogate for the *ab initio* calculations. We validate the performance of the developed MLIPs through a comparison of structural, vibrational, and diffusive properties of liquid water with *ab initio* results. Our scheme offers tailored MLIPs that are data-efficient, accurate, and fast for molecular dynamics.

## Methods

### Hybrid Functional Calculations

Throughout this work, our target system consists of a periodic model of water molecules described at the hybrid functional level. Since foundation models are generally developed at the semilocal generalized gradient approximation (GGA) or the meta-GGA level, the choice of this target system highlights the need for tailored MLIPs. The water model contains 64 H_2_O molecules in a cubic box with a side of 12.42 Å, resulting in a density of 0.9998 g/cm^3^. As initial configuration, we take the last structure from a previous molecular dynamics trajectory.^32^ This trajectory was obtained from a 10 ps long NVT simulation at 350 K, based on the rVV10 functional.^33,34^

We perform all the hybrid functional calculations using the open-source CP2K code with the QuickStep module.^35^ We use the hybrid functional introduced in ref 36. This functional is based on the hybrid functional PBE0 (α),^37^ in which the fraction of Fock exchange (α) is set to 0.40. The dispersion forces are described by the nonlocal electron correlation functional rVV10,^33,34^ in which the parameter *b* is set to 5.3 to reproduce the experimental density of liquid water.^38^ We adopt the double-ζ valence basis set (DZVP), with an auxiliary plane wave cutoff of 600 Ry.^39,40^ The core–valence interactions are described by Goedecker–Teter–Hutter pseudopotentials.^41^ The self-consistent optimization of the wave functions is performed using orbital transformation (OT) as implemented in CP2K, with a convergence threshold of 1 × 10^–5^ a.u.^42^

We note that the adopted reference functional can lead to significant variations in the predicted water properties, such as the water density.^43,44^ Our choice of using a hybrid functional with a mixing parameter of α = 0.40 augmented with the rVV10 functional leads to a balanced description of the experimental structural, dynamical, and electronic properties of liquid water.^36,45^ Various fractions of exact exchange have been used in the literature to describe liquid water. Todorova et al. used a fraction of exact exchange equal to 0.25 and achieved reasonable structural and dynamic properties.^46^ Gaiduk et al. used the inverse of the high-frequency dielectric constant of liquid water (1.77), corresponding to a fraction of exact exchange equal to 0.565 and also found good agreement with experimental RDFs.^47^ These findings indicate that reasonable descriptions of structural and dynamical properties can be obtained for a large range of exact-exchange fractions. As far as the electronic properties are concerned, Bischoff et al. show that lowering the amount of exact exchange results in an underestimation of the band gap, while increasing the exact exchange to around 60% leads to a band gap of 10 eV, higher than the experimental value of 9.2 ± 0.2 eV.^48^ Recently, it has been shown that hybrid functionals satisfying the piecewise linearity condition give electronic band gaps as accurate as many-body perturbation calculations.^48–50^ Our setup results in a band gap of 8.75 eV,^36^ close to the experimental range although still slightly underestimated. The dielectric constant decreases with an increasing fraction of exact exchange due to the increasing band gap. However, systematic studies of the dielectric constant variation with the mixing parameter are unavailable in the literature. In ref 48, it is shown that a range-separated hybrid functional with a mixing parameter of 0.57 in the long range yields a dielectric constant of 1.74, in good agreement with the experimental value of 1.77.

In the AIMD, we set the temperature to 350 K to ensure a frank diffusive motion of the water molecules.^36^ The temperature is controlled by a Nosé–Hoover chain thermostat with a time constant of 0.1 ps. The equations of motion are integrated using the velocity Verlet algorithm with a time step of 0.5 fs. In this way, the molecular dynamics simulation samples the canonical ensemble at a fixed volume and number of particles (NVT). We run the AIMD simulations for a duration of 10 ps, which is sufficient to obtain structural, vibrational, and diffusive properties.^36^

### Machine Learning Interatomic Potentials

In this section, we briefly describe the descriptor-based DeePMD and graph-based MACE architecture. Readers are directed to the original publications for a more comprehensive description of the underlying theories.^7,51,52^

DeePMD is a descriptor-based method. The total energy is decomposed into a summation of individual atomic energies:^3^

1 $$E=\overset{N}{\underset{i}{\sum }}E_{i}$$

where *N* is the total number of atoms in the system and *E*_*i*_ is the site energy, which is described by the local chemical environment. The atomic positions are first mapped to two-body or three-body atomic representations ***D***_*i*_ to preserve the translational, rotational, and permutational invariance. These representations are denoted as descriptors, which are subsequently fed to the deep neural network.^52^ The input data pass through several connected hidden layers, undergoing linear and nonlinear transformations before being mapped to the site energy:

2 $$E_{i}=N\left(D_{i}\right)$$

where *N* denotes the neural-network mapping. For the training of the DeePMD model, the hyperparameters are set to commonly adopted values.^53,54^ We use a cutoff radius of 6 Å. The embedding and fitting neural networks contain three layers each, with (25, 50, 100) and (240, 240, 240) neurons, respectively, following previous work.^53,54^ The initial learning rate is set to 0.001 with a decay step of 2000. During the training, the energy prefactor of the loss function increases from 0.02 to 2, while the force prefactor decreases from 1000 to 1. The model is optimized with 400,000 steps with the Adam method.^53^ We perform the DeePMD model training using DeePMD-Kit v2.2.9.^55^

MACE has a message-passing graph neural network architecture, where the atoms are represented by nodes and the edges denote the connections between atoms within a certain cutoff radius. The node features (***h***) are learnable, and the high body-order representations can be conveniently constructed by increasing the message-passing layers or body order. In MACE, the atomic energy is obtained through a learnable combination of the site energies:^7^

3 $$E_{i}=\overset{S}{\underset{s=1}{\sum }}E_{i}^{s}=\overset{S}{\underset{s=1}{\sum }}R^{\left(s\right)}\left(h_{i}^{\left(s\right)}\right)$$

where the readout function *R* is linear for *s*  <  *S* and corresponds to a one-layer multilayer perceptron in the last layer (*s* = *S*). The atomic energy is then summed up to get the total energy as in DeePMD. In the training of the MACE potential, we set the cutoff radius to 6 Å. We set the correlation order to 3, corresponding to 4-body messages in each layer. Two message-passing layers (*L* = 2) are used to balance accuracy and efficiency, with 128 channels for tensor decomposition, as in previous work on ice.^23^ We set the batch size *N*_batch_ to 5. In each epoch, the entire data set is used in the training. The gradients are updated *N*/*N*_batch_ times, with *N* being the number of configurations in the training data. Therefore, the total number of optimization steps is given by *N*_epoch_ × *N*/*N*_batch_, where *N*_epoch_ is the number of epochs. Here, we set *N*_epoch_ to 500. As the foundation model, we take mace-omat-0-medium.model,^9^ which is trained on a data set produced with a semilocal density functional.^56^ Unless otherwise specified, the foundation model in the current work always refers to this model. For all the trainings, we use version 0.3.14 of MACE.

We use the code Large-scale Atomic/Molecular Massively Parallel Simulator (LAMMPS) to perform molecular dynamics simulations with either DeePMD or MACE models.^57^ In LAMMPS, the same NVT ensemble as that in the AIMD is adopted. We use the MACE models in LAMMPS with Kokkos and ML-IAP packages for better performance.^58^

### Active Learning

To obtain accurate MLIPs, the training data set needs to cover the desired region of the potential energy surface. Active learning workflows allow one to expand the training data set incrementally, thereby creating a comprehensive data set to reduce out-of-distribution errors. The active learning workflow is illustrated in Figure 1 and generally involves three stages:

1 **Labeling**—Accurate energy and forces for various structures are obtained to be added to the data set. Here, energies and forces are obtained through single-point calculations at the hybrid functional level. At the beginning, a small initial data set is provided to initialize the training.
2 **Training**—Machine learning models encode the structural information into energy and atomic forces. A committee of four models (*M*_1_, *M*_2_, *M*_3_, *M*_4_) is trained on the same data set with independently initialized parameters, and their predictions are used in the next stage to estimate the MLIP errors.
3 **Exploration**—Classical MD simulations are performed at a low cost using the models from Stage 2, which are more efficient than AIMD in sampling the potential energy surface. Then, the model deviation $\epsilon_{F}^{\max}$ of the committee models is computed as a surrogate for the real error. In this work, the model deviation is evaluated per frame using the atomic forces ***F***_*i*_. Configurations with model deviations in a defined range are sent to Stage 1 for labeling, as this indicates low model confidence.

**Figure 1:**
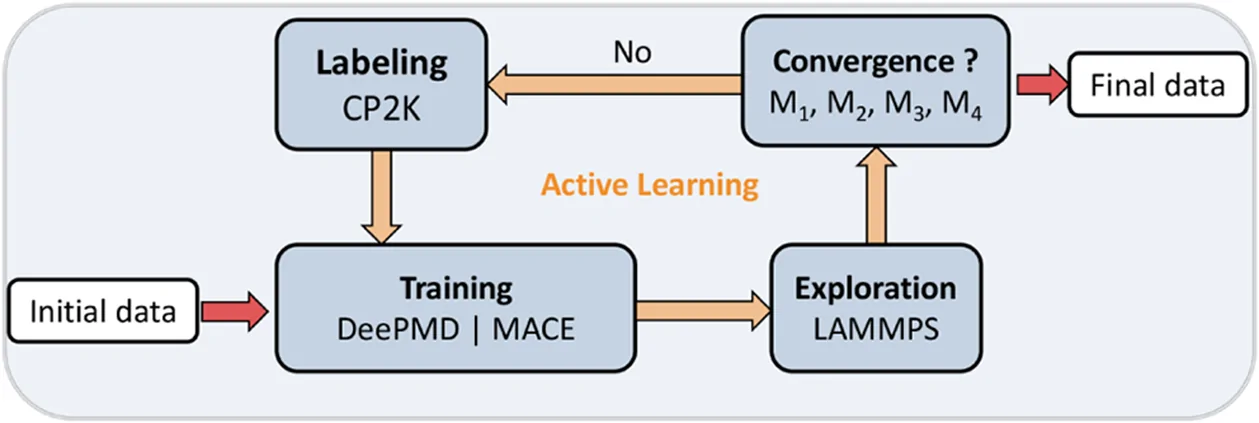
Scheme of the active learning workflow. The training can be conveniently done with either DeePMD or MACE, while exploration and labeling are accomplished with the classical MD code LAMMPS and the *ab initio* code CP2K, respectively.

The model deviation in the exploration stage is crucial for the active learning workflow and has been well established for DeePMD and implemented in the DP-GEN package.^59^ We have implemented the same method as an external postprocessing package for MACE. The relevant code is available in the GitHub repository.^60^ Here, we provide a brief introduction to the underlying theory. The committee average of the forces for the four models is computed as

4 $$\langle F_{i}\left(x;M_{k}\right)\rangle_{k}=\frac{1}{4}\overset{4}{\underset{k=1}{\sum }}F_{i}\left(x;M_{k}\right)$$

where ***x*** denotes the frame containing all coordinates and chemical species of all atoms, *i* denotes the index of an atom, and *M*_*k*_ refers to the models initialized with different parameters. Then, the model deviation for a given frame is defined as

5 $$\epsilon_{F}^{\max}\left(x\right)=\underset{i}{\max}\sqrt{{\left\langle {∥F_{i}\left(x;M_{k}\right)-\langle F_{i}\left(x;M_{k}\right)\rangle_{k}∥}^{2}\right\rangle }_{k}}$$

where *i* runs over all atoms of the frame. A large value of $\epsilon_{F}^{\max}$ indicates that the models describe the configuration with low confidence, and the corresponding frame is added to the training set after labeling. Otherwise, the models are assumed to be confident in their predictions, and the labeling is skipped. Previous work has demonstrated that the committee error shows a strong correlation with the real error.^18,59^ Therefore, configurations can be selected on the basis of these predictive errors after defining a threshold for the model deviation.

We perform active learning using the ai2-kit code.^61^ We create a very small initial data set of liquid water containing only 10 configurations sampled 100 fs apart from the AIMD trajectory. In practice, we set lower and higher bounds for the model deviation: σ_low_ = 0.1 eV/Å and σ_high_ = 0.8 eV/Å. The higher bound allows us to discard frames that are assumed to be unrealistic. We then run exploration tasks at 350, 450, and 500 K at each iteration, aiming to diversify the sampled data set. Our exploration tasks gradually increase from 0.5 to 25 ps, and 10 configurations are sampled at each iteration for labeling. We intentionally sample a small subset per iteration to follow the improvement gradually. All the labeling and exploration tasks are performed with CP2K and LAMMPS, except in the distillation process, where the fine-tuned foundation model is used for labeling.

All of the calculations in this work are performed at CSCS Daint@Alps, where each compute node consists of 4 GH200 modules equipped with CUDA 12.0. Each module pairs an NVIDIA Grace CPU with an H100 GPU. The *ab initio* calculations with CP2K are performed with the four modules. A single module is used for training with either DeePMD-Kit or MACE and for the MD simulations with LAMMPS.

## Results and Discussion

### MLIPs from Scratch

In this section, we compare the convergence of active learning and the computational speed of the DeePMD and MACE models. The two MLIPs are generated from scratch. Hence, this comparison highlights intrinsic differences resulting from the architectures of the models.

In Figure 2a, we show the evolution of the model uncertainty ratio during the active learning workflow. Here, we define *R*_good_ as the ratio between configurations with predicted errors below the lower threshold σ_low_ (*N*_σ*<*σ_low__) and the total number of sampled configurations (*N*) at each iteration, i.e., *R*_good_ = *N*_σ*<*σ_low__/*N*. Therefore, all configurations with predictive errors higher than σ_low_ are considered uncertain. As the model gains confidence, the ratio increases and the uncertainty 1 – *R*_good_ decreases. In the ideal case, all the curves in Figure 2a decrease toward zero, meaning that we obtain MLIPs with vanishing predictive errors. For all the models, we stop the learning when, in three consecutive iterations, *R*_good_ is 97% or above. Then, the first MLIP that satisfies this criterion is used for subsequent investigation. At the beginning, *R*_good_ is low for both models. This is due to the small initial data set, leading to low predictive capability for the models. The number of cumulative *ab initio* calculations (*N*_*ab initio*_) vs iteration is shown in Figure 2b. With an increasing data set, we see that *R*_good_ for MACE increases much faster than that for DeePMD [cf. Figure 2a]. This result is not surprising since MACE has a higher generalization capability and learns the interactions with less data. Figure 2b shows that *N*_*ab initio*_ increases with the same slope for MACE and DeePMD, since each iteration involves 10 new configurations in both cases. MACE deviates from this rule in the last iteration because only 7 configurations are still found to be uncertain. DeePMD reaches convergence in 24 iterations, while MACE only needs 7 iterations. The corresponding structures sampled from DeePMD and MACE are 250 and 77, respectively. Hence, MACE requires only one-third as many *ab initio* calculations as DeePMD.

**Figure 2:**
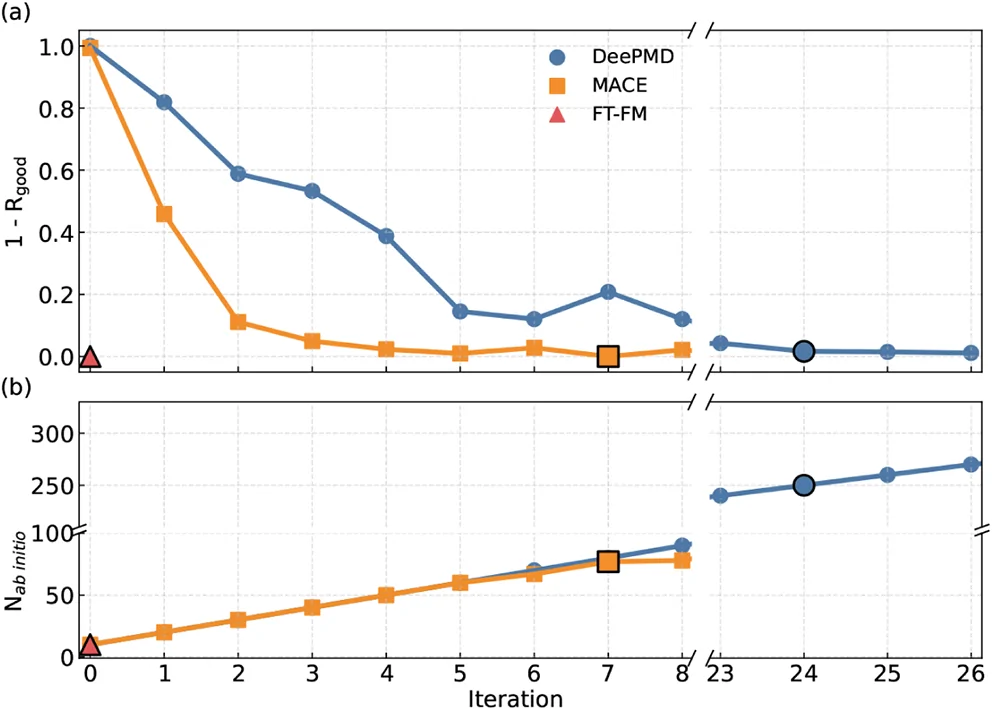
(a) Model deviation as a function of iteration during active learning. (b) Cumulative number of *ab initio* calculations *N_ab initio_* performed during active learning. The DeePMD, MACE, and FT-FM data are shown in blue disks, orange squares, and red triangles, respectively. The final converged iterations are highlighted with larger symbols with black edges. The axes are broken for visualization purpose, since DeePMD takes more iterations to converge.

The convergence of the active learning is closely related to the time cost; Table 1 summarizes the GPU time for the three stages. Since DeePMD takes more iterations to converge (Figure 2), the labeling time is around 3.2 times longer. The training stage of DeePMD costs significantly more than that of MACE, predominantly due to the number of training steps being larger by 3 orders of magnitude. In contrast, the exploration time shows a drastically different picture. Despite the fast convergence of model training with MACE, the exploration time exceeds that of DeePMD by a factor of 3, indicating a much slower inference speed (*vide infra*). When we consider the full time cost, we see that DeePMD takes two times longer to reach our defined convergence criteria.

**Table 1:** Analysis of Active Learning Time (in GPU Hours) for the Various MLIP Models Discussed in This Work^a^

Model	Labeling (*ab initio*)	Labeling (FT-FM)	Training	Exploration	Total
DeePMD	66.9	–	73.4	12.6	152.9
MACE	20.9	–	5.8	37.0	63.7
FT-FM	2.7	–	0.3	–	3.0
DistillM	2.7	0.3	11.7	1.5	16.2

a The cost is split into labeling, training, and exploration time. In the DistillM case, the labeling costs at the *ab initio* and FT-FM levels are given separately.

Figure 3 provides inference costs for DeePMD and MACE models, relevant for both the corresponding exploration stage of active learning and the subsequent MD simulations performed using the models. We consider system sizes with a number of atoms up to 12288. The DeePMD models consistently outperform the MACE models for all considered system sizes. This is a consequence of the more complex message-passing graph neural network of MACE. The inference speed of the DeePMD model is greater than that of the MACE model by a factor that decreases from 16 to 9 as the system size increases. Hence, DeePMD generally achieves a speed-up of about 10× or more, though this advantage over MACE declines for larger systems. Therefore, for calculations involving thousands of atoms or simulations requiring long time scales, such as for free-energy calculations, DeePMD carries a significant advantage over MACE as far as inference speed is concerned.^62^

**Figure 3:**
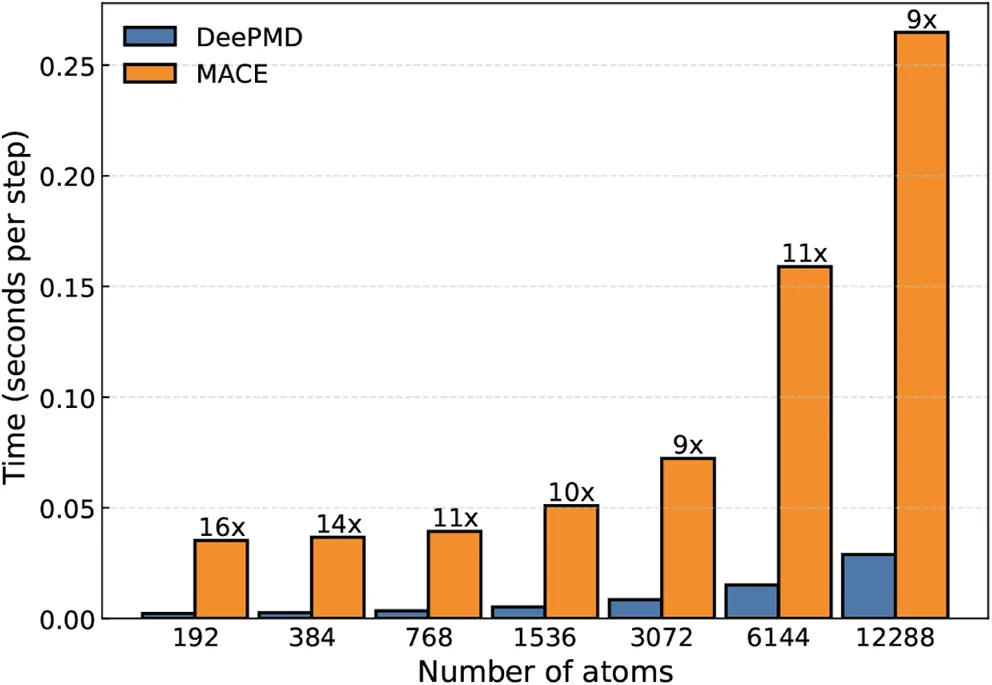
Computational time of MACE (orange bars) and DeePMD (blue bars) models as a function of the number of atoms in the liquid–water system. Tests are conducted on a single GH200 module, except for the systems with 6144 and 12288 atoms calculated with MACE. For these two systems, we use two modules due to memory issues, and the corresponding time is given per single module for a fair comparison. The ratio between the inference time per step of the two models is given for each system size.

### Strategies for Data-Cost Reduction and Faster Inference

The comparative study in the previous section provides important insights into the active learning cost and the inference efficiency of the two MLIP architectures. It is clear that computer time is saved when the training is performed with MACE, while DeePMD offers faster inference speeds once the MLIP is generated. In this section, we show how these advantages can be used together.

To further reduce the active learning time, we investigate the potential of foundation models based on the MACE architecture. Foundation models have been pretrained with large databases, suggesting that they could provide a convenient starting point for active learning. Therefore, we use the same active learning workflow introduced above to generate a fine-tuned foundation model (FT-FM). In Figure 2, the model deviation and the number of *ab initio* calculations as a function of iteration are compared to the DeePMD and MACE models trained from scratch. The corresponding time estimates are given in Table 1. We find that the foundation model fine-tuned on the initial 10 configurations cannot be further improved in the first iteration of the active learning workflow. Hence, the labeling cost in Table 1 corresponds to these initial configurations, and no further exploration is required. This result is consistent with previous work in the literature, showing that a small data set can already lead to satisfactory MLIPs for liquid water and ice.^23,31^ The use of the foundation model as a starting point reduces the total active learning time by 51 and 13 times compared with DeePMD and MACE, respectively. These remarkable data-cost reductions strongly favor fine-tuning a foundation model rather than training from scratch.

Additionally, to increase the inference speed, we developed a two-step knowledge distillation process leading to a DeePMD model (cf. Figure 4). This process integrates two sequential active-learning cycles. In the first cycle, we obtain the FT-FM as described above to ensure data efficiency. Next, we trained the DeePMD model using a second cycle of active learning. The FT-FM is used to compute the energy and forces for the data sampled in the exploration stage, rather than performing additional *ab initio* calculations. The resulting DeePMD model is denoted as DistillM.

**Figure 4:**
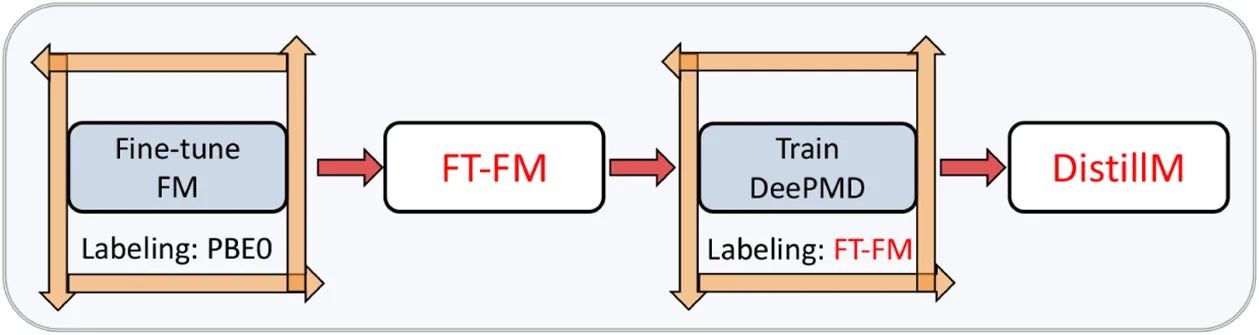
Scheme of the two-step distillation process involving two active learning cycles, leading to the distilled model (DistillM). In the first cycle, the foundation model is fine-tuned, leading to an accurate FT-FM. In the second cycle, a DeePMD model is achieved by active learning, where labeling is performed with the FT-FM generated in the first cycle.

The total cost for generating the distilled model is given in Table 1. Two labeling costs are distinguished. The first derives from the labeling in the first cycle performed at the *ab initio* level to generate the FT-FM, while the second corresponds to the labeling performed by the FT-FM in the second cycle. In the second cycle, the labeling stage involves a large number of configurations, but its cost is found to be negligible because no *ab initio* calculations are involved. The distilled model DistillM is found to converge in three iterations, each sampling at most 400 configurations. As can be seen in Table 1, the dominant part of the total cost arises from the training stage required for converging the DeePMD model. Furthermore, we remark that for calculations climbing up Jacob’s ladder or involving target systems of larger size, the labeling cost would increase drastically, and the benefit of our distillation scheme would be even more pronounced.^12,63^ In the present study, the cost associated with the two-step distillation process is four times lower than MACE and 10 times lower than DeePMD (cf. Table 1). In addition, our distilled model allows us to leverage the high inference speed of DeePMD models, making it possible to perform long-duration MD simulations, such as those required for the determination of free-energy surfaces.^64,65^

### Accuracy of MLIPs and Reproduction of Physical Properties

We evaluate the model performance during active learning via the root-mean-square errors (RMSEs) of the energy and atomic forces on an independent data set containing 500 configurations. The test set is obtained from a 100 ps NVT simulation using the MLIP from ref 65. We then calculate the *ab initio* energy and forces with the same hybrid functional used in the labeling stage of active learning. In Figure 5, we give the RMSEs of the energy and atomic forces for each iteration. We can see that, upon considering the 10 initial configurations, MACE already predicts a significantly lower error compared to DeePMD, leading to the rapid convergence of the MACE model. Additionally, the final RMSEs of energy and forces for MACE are around 3.5 and 2 times smaller than those of DeePMD, respectively, demonstrating the high accuracy of the MACE architecture. It is worth noting that the error of the DeePMD models cannot be decreased further even when extending active learning to 30 iterations. This suggests that the level of error is related to the model architecture rather than the training data set. Nevertheless, the accuracy achieved with DeePMD is close to previously reported values and sufficiently accurate for achieving an accurate description of several physical properties of the liquid–water system.^13,17,66,67^

**Figure 5:**
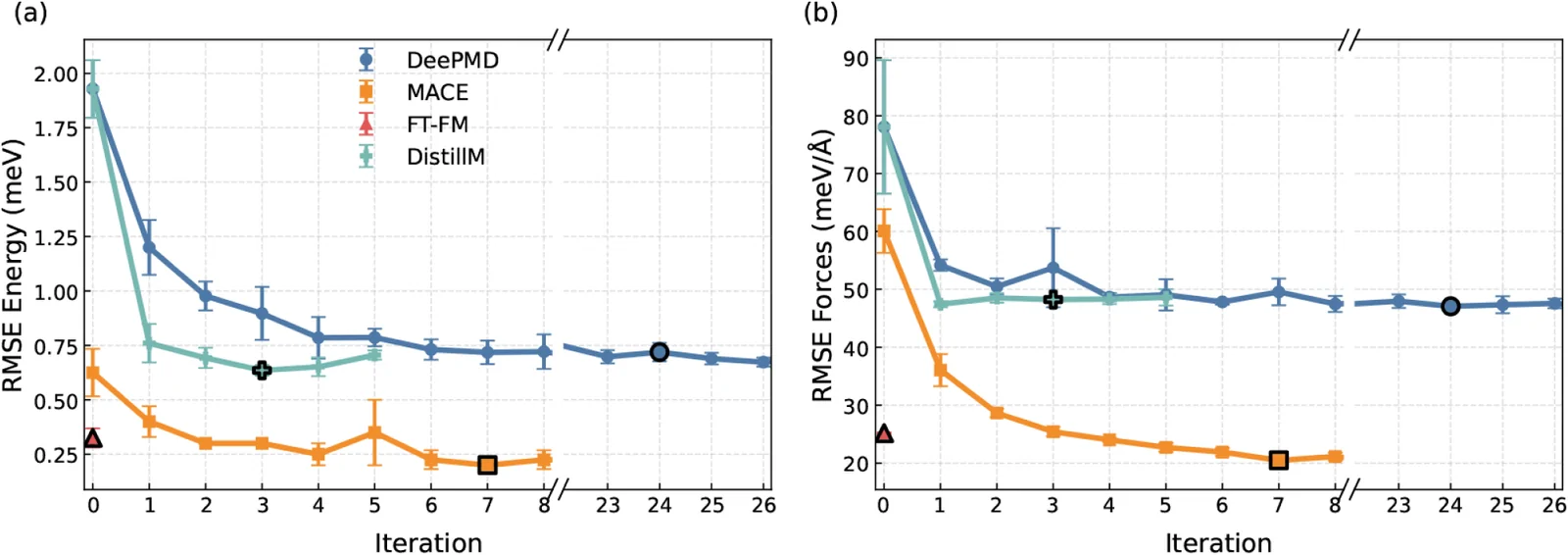
Root mean square errors (RMSE) as a function of iteration during active learning for (a) energy and (b) atomic forces. The colors and symbols are the same as in Figure 2. The distilled model is shown with cyan crosses. The error bars show standard deviations.

The fine-tuned foundation model reaches RMSE values similar to those of the MACE model trained from scratch, thereby further supporting the fine-tuning approach. The RMSEs of the distilled model achieve the same level of accuracy as that of the DeePMD model trained from scratch. This indicates that the FT-FM is accurate enough to label data in active learning and that the resulting models can perform as well as those labeled by using *ab initio* calculations.

The higher error of the DeePMD models relative to MACE has been attributed to a limitation of its invariant, descriptor-based representation.^68^ In contrast, including equivariance in the MLIP enables the learning of directional information and has been shown to more effectively capture many-body interactions,^69^ achieving better accuracy than invariant models.^68^ Many-body decomposition analysis of liquid water demonstrates that DeePMD models trained exclusively on liquid-phase configurations fail to adequately capture many-body interactions.^68–70^ In this work, we do not aim to address such limitations of DeePMD but focus on physical properties that can be well reproduced with this framework (*vide infra*).

To further assess the accuracy of the various MLIPs, we calculate the structural, vibrational, and diffusive properties for a liquid–water system of 64 molecules and compare them with their *ab initio* counterparts. Specifically, we focus on the O–O and O–H radial distribution functions, the power spectrum, and the diffusion constant.

To illustrate the benefit of tailored MLIPs, we start this comparison by assessing the performance of the original foundation model,^9^ for which the results are reported in Figure S1 of the Supporting Information (SI). As can be inferred from the O–O and O–H radial distribution functions [Figure S1a and b], the foundation model produces a satisfactory agreement with the *ab initio* results, albeit the water structure is found to be slightly enhanced. However, the descriptions of the power spectrum [Figure S1c] and diffusivity [Figure S1d] obtained with the foundation model differ noticeably from our hybrid functional results. These discrepancies stem from the fact that the adopted foundation model is based on a semilocal functional.^9^ This supports the need for tailored MLIPs when targeting theories of higher levels.

Figure 6 shows the calculated physical properties for the various MLIPs developed in this work in comparison with our hybrid functional results. The structural and vibrational properties obtained with the MLIPs correspond to averages over 1 ns. As shown in Figure 6a and b, the oxygen–oxygen and oxygen–hydrogen radial distribution functions of all four MLIPs accurately reproduce the AIMD results. The first peak position of the oxygen–oxygen radial distribution function is found at 2.72 Å in the AIMD and is correctly reproduced in all the MLIP-based simulations. This is consistent with the experimental peak position at 2.80 Å.^71^ The MLIPs also correctly reproduce the height of the first peak found in the AIMD, which is slightly higher than the experimental peak height. In the SI, Table S2 summarizes the peak position, the height, and the full width at half-maximum (fwhm) of the first peak in the oxygen–oxygen and oxygen–hydrogen radial distribution functions found with the AIMD and MLIP-based MD simulations, along with corresponding experimental data.

**Figure 6:**
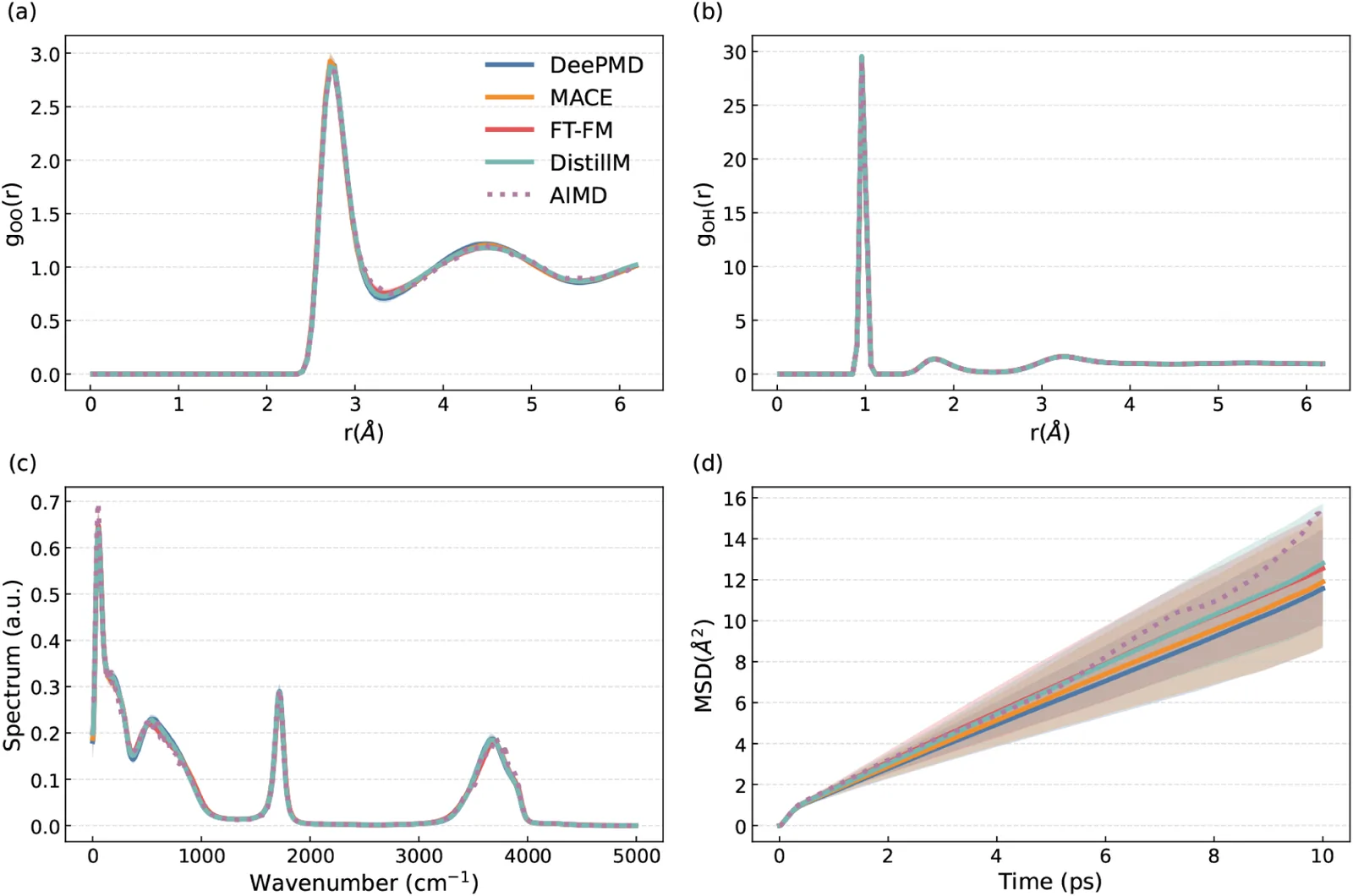
(a) Oxygen–oxygen radial distribution function *g*_OO_. (b) Hydrogen–hydrogen radial distribution function *g*_OH_. (c) Total power spectrum of liquid water. (d) Oxygen mean square displacement (MSD) vs time. The results correspond to a liquid–water system consisting of 64 water molecules and are obtained with DeePMD (blue), MACE (orange), FT-FM (red), DistillM (cyan), and AIMD (purple dotted). For the MLIPs, the shaded areas indicate standard deviations estimated from 100 independent simulations with different initial velocities.

Next, we focus on the vibrational properties, which correlate with the description of the hydrogen bond network.^72–74^ We calculate the power spectrum from the Fourier transform of the velocity autocorrelation function. The four MLIPs developed in this work produce almost identical peak positions and intensities to those found with AIMD [cf. Figure 6c], demonstrating their ability to properly capture the hydrogen bond network. The peak positions and the fwhm of the power spectrum are provided in Table S3 of the SI. More specifically, the AIMD simulation yields peak positions at 3705 cm^–1^, 1718 cm^–1^, and 584 cm^–1^, corresponding to the O–H stretch, H–O–H bend and the librations, respectively, which should be compared with the experimental peaks at 3404 cm^–1^, 1644 cm^–1^, and 400–800 cm^–1^.^75–77^ The H–O–H bending and O–H stretching are blue-shifted from the experimental peaks by 9% and 5%, respectively, with the libration peak lying well within the experimental range.

Figure 6d shows the mean square displacements of the oxygen atoms as a function of time, as obtained with the four MLIPs. The curves correspond to averages over 100 independent simulations, while the shaded areas reflect the standard deviations. These evolutions can be compared with our hybrid functional result, which corresponds to a single trajectory of 10 ps. The corresponding diffusion coefficients *D* are obtained from Einstein’s relation and are given in Table 2. All the MLIPs yield consistent diffusion coefficients. From the single AIMD trajectory, we obtain a diffusion coefficient of 2.0 × 10^–5^ cm^2^/s, which is also consistent with the four MLIPs. This suggests that the MLIPs properly reproduce the diffusive behavior of the hybrid functional. The calculated diffusion constants compare well with the experimental value at 300 K after applying the Yeh–Hummer finite-size correction pertaining to the simulation cell (cf. Table 2).^36,79^

**Table 2:** Self-Diffusion Coefficient *D* as Obtained from AIMD and MD Simulations Performed Using DeePMD, MACE, FM, and DistillM^a^

Method	*D* (cm^2^/s)
AIMD	2.0 × 10^–5^
DeePMD	(1.9 ± 0.5) × 10^–5^
MACE	(2.0 ± 0.5) × 10^–5^
FT-FM	(2.2 ± 0.5) × 10^–5^
DistillM	(2.1 ± 0.4) × 10^–5^
Experimental	1.8 × 10^–5^

a Errors correspond to standard deviations obtained from 100 independent simulations. We have added the experimental diffusivity at 300 K after finite-size correction.^77,78^

Therefore, when we globally consider all of the physical properties studied here, we conclude that all MLIPs exhibit similar quality, essentially with no significant loss of accuracy compared to the *ab initio* results. This conclusion is particularly relevant for the fine-tuned foundation model obtained with just 10 labeled configurations and even more so for the distilled model, which combines this performance with high inference speed.

In addition to the physical properties discussed above, we also checked to what extent our MLIPs correctly reproduce the density of liquid water at 350 K under atmospheric pressure. Our results in the SI show that the density is a more delicate physical property that cannot be accurately obtained without dedicated training data. Indeed, inclusion of such training data restores the good agreement for all MLIPs,^80^ showing that the present methodological framework preserves its validity also for this property when it is properly addressed. Similarly, reproducing delicate properties, such as the melting temperature and nuclear quantum effects on either the density maximum or melting temperature, requires the generation of devoted MLIPs.^81,82^

## Conclusions

In this work, we develop data-efficient MLIPs for performing fast molecular dynamics by combining the relative advantages of descriptor-based and graph-based architectures. More specifically, we leverage the data efficiency of MACE and the high inference speed of DeePMD.

Using liquid water described at the hybrid functional level as the target system, we generate DeePMD and MACE models from scratch. DeePMD requires three times more training data than MACE when using active learning but yields inference speeds that are about a factor of 10 faster. To enhance data efficiency, we turn to a pretrained MACE foundation model, which substantially reduces the number of required *ab initio* calculations and supports fine-tuning as a viable approach.

Then, we develop a two-step distillation process combining two cycles of active learning workflows. The first cycle generates an accurate fine-tuned foundation model with minimal *ab initio* calculations. In the second cycle, this model is used to label data in the active learning of a DeePMD model. The two-step process is about 10 times faster than training DeePMD models from scratch and yields a distilled model showing the same high inference speed. All developed potentials are validated against *ab initio* results for energies and atomic forces on an independent data set. A comparison of physical properties confirms that all models reproduce the *ab initio* reference without any loss of accuracy. In conclusion, the present two-step process enables data-efficient and fast machine-learning molecular dynamics, is broadly applicable across diverse systems, and advances the development of tailored MLIPs for domain-specific applications. In particular, this approach also represents a preliminary step toward the description of electric and/or electronic properties.^83–85^

## Supplementary Material



## Data Availability

The data underlying this study are available in the published article and the Supporting Information. The scripts and code used to generate and analyze the results are available at the Materials Cloud archive: 10.24435/materialscloud:bk-23.

## Notes

Dataset size of the MLIPs; physical properties obtained with the foundation model; first peaks of radial distribution function; vibrational normal modes of power spectrum; density of liquid water from MLIPs at 350 K; (PDF)
