# Supplementary material for: Data-Efficient and Fast Machine Learning Molecular Dynamics through Integrated Active Learning and Knowledge Distillation
Source: J Chem Theory Comput. 2026 Aug 21;22(17):8730–9. doi: 10.1021/acs.jctc.6c00917 (PMC13564361; doi:10.1021/acs.jctc.6c00917)
Supplement: Supplementary file 1 [file ct6c00917_si_001.pdf]

Data-Efficient and Fast Machine Learning Molecular Dynamics through Integrated Active Learning and Knowledge Distillation

Xiliang Lian* and Alfredo Pasquarello

Chaire de Simulation à l'Echelle Atomique (CSEA), Ecole Polytechnique Fédérale de Lausanne (EPFL), CH–1015 Lausanne, Switzerland

E-mail: xiliang.lian@epfl.ch

Dataset size of the MLIPs

In Table S1, the number of structures in the final dataset for each model is given. We distinguish those labeled by *ab initio* from those labeled by FT-FM for the distilled model. DeePMD, MACE, and FT-FM are fully trained/fine-tuned with *ab initio* data, which DistillM is obtained with data labeled by FT-FM except for the initial dataset.

Table S1: Dataset size of various MLIP models discussed in this work.

Model	*ab initio*	FT-FM
DeePMD	250	–
MACE	77	–
FT-FM	10	–
DistillM	10	1712

Physical properties obtained with the foundation model

In Figure S1, physical properties obtained with the original foundation model (FM), mace-omat-0-medium.model,[1] are compared with results from FT-FM and AIMD given in the main text. The diffusion constant for the foundation model is 0.9×10^{-5} cm^2/s.

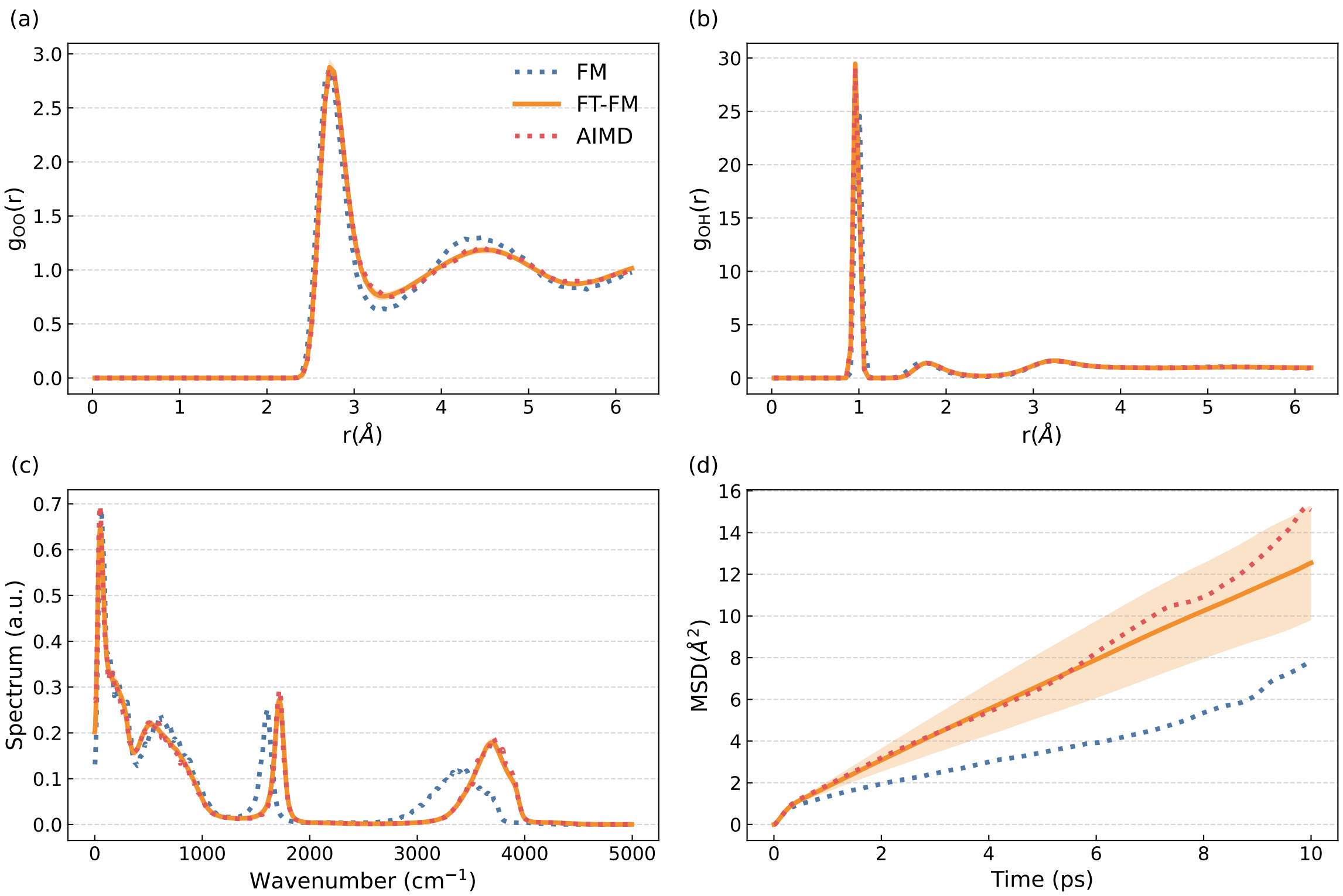

Figure S1: (a) Oxygen-oxygen radial distribution function g_{OO}. (b) Oxygen-hydrogen radial distribution function g_{OH}. (c) Total power spectrum of liquid water. (d) Oxygen mean square displacement (MSD) vs. time. The results correspond to a liquid-water system of 64 water molecules and are obtained with the original foundation model (FM), mace-omat-0-medium.model (cyan), the fine-tuned foundation model, the FT-FM (orange), and the AIMD (blue). The diffusion constant for the foundation model is 0.9×10^{-5} cm^2/s. For the FT-FM, the shaded area indicates standard deviations estimated from 100 independent simulations with different initial velocities.

First Peaks of Radial Distribution Function

In Table S2, first peak positions, heights, and full width at half maximum (FWHM) of the oxygen-oxygen ($g_{OO}(r)$) and oxygen-hydrogen ($g_{OH}(r)$) radial distribution functions are summarized. Experimental results are summarized from ref 2–4.

Table S2: First peak positions, heights, and full width at half maximum (FWHM) of the oxygen-oxygen ($g_{OO}(r)$) and oxygen-hydrogen ($g_{OH}(r)$) radial distribution functions.

	$g_{\mathrm{OO}}(r)$ first peak			$g_{\mathrm{OH}}(r)$ first peak		
**Method**	Position (Å)	Height	FWHM (Å)	Position (Å)	Height	FWHM (Å)
AIMD	2.72	2.83	0.31	0.96	29.15	0.08
DeePMD	2.72	2.92	0.31	0.96	29.42	0.08
MACE	2.72	2.93	0.30	0.96	29.30	0.08
FT-FM	2.72	2.88	0.31	0.96	29.46	0.08
DistillM	2.72	2.88	0.31	0.96	29.54	0.08
Experimental[2–4]	2.80	2.58	0.31	0.97	31.80	0.05

Vibrational Normal Modes of Power Spectrum

Table S3 shows the peak positions and FWHM of the power spectrum of liquid water obtained with different MLIPs. The experimental results are summarized from ref 2,5,6.

Table S3: Peak positions and full width at half maximum (FWHM) from the power spectrum of liquid water.

	Libration (cm^{-1})		H-O-H bend (cm^{-1})		O-H stretch (cm^{-1})	
**Method**	Position	FWHM	Position	FWHM	Position	FWHM
AIMD	584.31	177.24	1718.31	82.46	3705.36	378.54
DeePMD	561.91	263.8	1718.31	90.22	3678.90	387.21
MACE	519.16	234.55	1718.31	95.31	3689.08	396.3
FT-FM	529.34	232.83	1716.28	97.64	3691.11	405.58
DistillM	543.59	244.05	1716.28	91.61	3674.82	390.28
Experimental[2,5,6]	400-800	–	1644	–	3404	–

Density of Liquid Water from MLIPs at 350 K

Table S4 reports the liquid water density obtained from 50-ps NPT simulations at 350 K and atmospheric pressure using the different MLIPs. The errors are estimated from 10 independent simulations, summing up to 500 ps. ρ_1 is obtained using the original dataset collected in the current work. While the DeePMD and DistillM models give reasonable agreement with the AIMD result, both MACE and FT-FM overestimate the water density. The overestimation might result from the fact that getting the correct density requires a dedicated dataset to account for the potential energy surface variation.[7,8] Indeed, our training data have not specifically been designed to account for volume variation. To solve this issue, we use the dataset from ref 9, which is obtained at the same level of reference theory (ρ_2). All MLIPs demonstrate excellent agreement with the reference *ab initio* density.

Table S4: Densities (at 1 atm and 350 K) obtained from MD simulations performed using DeePMD, MACE, FT-FM, and DistillM. ρ_1 and ρ_2 correspond to the densities obtained from the models trained on the original small dataset in the manuscript and the dataset from ref 9, respectively. These values should be compared with the reference AIMD result of 1.02 g/cm^3 from ref 10.

Method	ρ_1 (g/cm^3)	ρ_2 (g/cm^3)
DeePMD	1.00±0.01	1.02±0.01
MACE	1.15±0.00	1.01±0.01
FT-FM	1.22±0.00	1.04±0.01
DistillM	1.05±0.00	1.01±0.01

References

(1) Batatia, I. et al. A Foundation Model for Atomistic Materials Chemistry. *J. Chem. Phys.* **2025**, *163*, 184110.

(2) Gittus, O. R.; Bresme, F. Thermophysical Properties of Water Using Reactive Force Fields. *J. Chem. Phys.* **2021**, *155*, 114501.

(3) Skinner, L. B.; Huang, C.; Schlesinger, D.; Pettersson, L. G. M.; Nilsson, A.; Benmore, C. J. Benchmark Oxygen-Oxygen Pair-Distribution Function of Ambient Water from x-Ray Diffraction Measurements with a Wide Q-range. *J. Chem. Phys.* **2013**, *138*, 074506.

(4) Dong, R.-K.; Mei, Z.; Zhao, F.-Q.; Xu, S.-Y.; Ju, X.-H. Molecular Dynamics Simulation on the Reaction of Nano-Aluminum with Water: Size and Passivation Effects. *RSC Adv.* **2019**, *9*, 41918–41926.

(5) Venyaminov, S. Y.; Prendergast, F. G. Water (H_2O and D_2O) Molar Absorptivity in the 1000–4000 Cm $^{-1}$ Range and Quantitative Infrared Spectroscopy of Aqueous Solutions. *Analytical Biochemistry* **1997**, *248*, 234–245.

(6) Max, J.-J.; Chapados, C. Isotope Effects in Liquid Water by Infrared Spectroscopy. III. H_2O and D_2O Spectra from 6000tocm-1. *J. Chem. Phys.* **2009**, *131*, 184505.

(7) ONeill, N.; Shi, B. X.; Baldwin, W. J.; Bartok, A. P.; Pickard, C. J.; Michaelides, A.; Csanyi, G.; Berkelbach, T. C. How Reproducible Are First-Principles Simulations of Liquid Water? 2026; DOI: 10.48550/arXiv.2605.28798.

(8) Kim, M.; Kim, S.; Jung, J.-Y.; Ha, M. Y.; Lee, W. B. Density Diversity in Training Data Governs Thermodynamic Transferability of Machine Learning Interatomic Potentials. 2026; DOI: 10.48550/ARXIV.2605.05733.

(9) Lan, J.; Rybkin, V. V.; Pasquarello, A. Temperature Dependent Properties of the Aqueous Electron. *Angew. Chem. Int. Ed.* **2022**, *61*, e202209398.

(10) Ambrosio, F.; Miceli, G.; Pasquarello, A. Structural, Dynamical, and Electronic Properties of Liquid Water: A Hybrid Functional Study. *J. Phys. Chem. B* **2016**, *120*, 7456–7470.
